# Construction and Validation of a Specific Balance Test for Rhythmic Gymnasts: A Pilot Study

**DOI:** 10.3390/sports14050178

**Published:** 2026-04-29

**Authors:** Rebeka Stojkovic, Ivan Peric, Zvonimir Tomac, Hrvoje Ajman, Zoran Spoljaric

**Affiliations:** Faculty of Kinesiology, University of Josip Juraj Strossmayer in Osijek, Drinska 16a, 31000 Osijek, Croatia; rstojkovic@kifos.hr (R.S.); ztomac@kifos.hr (Z.T.); hajman@kifos.hr (H.A.); zspoljaric@kifos.hr (Z.S.)

**Keywords:** rhythmic gymnastics, balance assessment, construct validity, reliability, sport-specific testing

## Abstract

Background: Balance is a key ability in rhythmic gymnastics, affecting not only technical performance but also the aesthetic and expressive quality of routines. Standard tests often do not reflect the real demands of the sport, where gymnasts must simultaneously maintain stability, manipulate apparatus, and follow the musical rhythm. Therefore, there is a need for a specific test that combines motor and cognitive challenges to provide a precise and reliable assessment of athletes’ functional abilities. Methods: The study involved 12 girls with an average age of 9 years. Participants underwent anthropometric measurements and were tested using standard motor tests as well as a specific balance test for rhythmic gymnasts (BTRG). Test reliability was assessed using a test–retest procedure, and construct validity was evaluated through factor analysis in comparison with existing balance tests. Results: The BTRG demonstrated high reliability (ICC = 0.96; CV = 6.4%; SEM = 0.18) and the ability to distinguish gymnasts from different programs. Factor analysis confirmed that the BTRG effectively measures specific balance in accordance with theoretical expectations. Conclusions: The new test provides a potentially valid and reliable tool for assessing specific balance in rhythmic gymnasts and maybe useful in the training process, athlete evaluation, and talent development; however, these finding should be interpreted with caution as they are preliminary and derived from pilot study.

## 1. Introduction

Rhythmic gymnastics is a conventional sport in which balance, as a motor ability, occupies one of the highest positions in the performance specification equation [1]. Beyond technical execution, balance contributes to the aesthetic and expressive quality of rhythmic gymnastics performance. In the current Olympic cycle, the values of body elements that require a highly developed ability to maintain balance have increased markedly, as defined in the Rhythmic Gymnastics Code of Points [2]. Balance is divided into static and dynamic form in the context of movement. Static balance refers to the ability to maintain a stable body position without changing the base of support, that is, without moving one or both legs of the body. In contrast, dynamic balance implies maintaining stability during movement, where disruption of the balance position inevitably occurs due to changes in the position of the body in space. Specific balance maintenance relies on information from several main sources.

The first consists of somatosensory information originating from mechanoreceptors in the feet, which enables the perception of the body weight distribution. The second and the third source comprises information from the vestibular and visual systems, which together provide essential input for spatial orientation and the perception of body position and movement [3]. The ability to maintain balance and postural stability is the result of a complex integration of various neuromuscular factors, with a key role played by the central processing of multisensory information from the vestibular and visual systems, as well as proprioceptive inputs from the periphery [4]. Balance at the level of the feet refers to the ability to maintain the vertical projection of the body’s center of mass within the base of support. Postural control mechanisms, resulting from the natural dynamics of a living organism, continuously adjust the body axis, leading to small and constant oscillations of the body during an upright standing position. These oscillations play an important role in the distribution of pressure on the foot.

Balance assessment is a complex process, and stabilometric analysis is one of the most commonly used research methods for measuring postural control. This method involves the use of force platforms capable of identifying the neuromuscular and biomechanical strategies employed to maintain balance in different directions of movement. The most frequently used stabilometric parameter in such assessments is the area of displacement of the center of pressure (COP), defined as the point of application of the resultant vertical forces acting on the support surface of the participant. As COP displacements are representative of postural sway, they are recorded through continuous calculation of position in the x and y coordinates, corresponding to movements in the anteroposterior and mediolateral directions [5]. Previous research has emphasized the critical role of balance in rhythmic gymnastics and has examined it extensively. For example, the Sensory Organization Test (SOT) has been applied to compare the abilities of rhythmic gymnasts and their peers under conditions involving different somatosensory, visual, and vestibular stimuli [6]. The results showed that rhythmic gymnasts were superior in terms of physical fitness and were more effective at maintaining balance when visual information was unreliable. Although adolescent rhythmic gymnasts demonstrated a similar learning rate to their peers, they exhibited better sensory organization abilities and adapted more quickly to a reduced reliance on visual input in demanding conditions. Also, previous research has consistently highlighted the importance of balance as a key performance related ability in rhythmic gymnastics, with a particular focus on dynamic balance and its underlying neuromuscular and sensory mechanisms.

Various methodological approaches have been employed to assess balance performance in this population, ranging from sport-specific field tests to instrumented posturographic measurements (PMS). Sport-specific dynamic balance assessments are frequently based on controlled transitions between characteristic body positions. A valid testing protocol has been proposed involving repeated transitions from passé to arabesque performed with both dominant and non-dominant legs. Performance is quantified by the number of correctly executed repetitions until postural alignment is disrupted or balance is lost, highlighting the importance of technical control and postural stability under sport-specific conditions [7]. In addition to field-based assessments, PMS are commonly used to evaluate the dynamic balance of rhythmic gymnasts. Over a two-year training period, longitudinal observations have tracked changes in dynamic balance and somatic characteristics in young gymnasts using statokinesiometric posturography [8]. Participants were required to control their center of mass within a continuously moving virtual target displayed on a monitor, allowing for controlled displacement of posture in multiple directions. The authors reported superior performance during the return from the target to the starting position, suggesting an enhanced capacity for rapid postural correction and stabilization developed through systematic training.

Overall, existing research indicates that rhythmic gymnasts exhibit superior dynamic balance compared with non-trained peers, which appears to result from sport-specific technical demands, enhanced postural control strategies, and adaptations in sensory integration and joint mobility. However, despite the diversity of assessment methods, there remains a need for standardized, sport-specific balance tests with clearly established validity and reliability to further advance performance evaluation and talent development in rhythmic gymnastics. Nevertheless, most available tests assess only general static or dynamic balance under conditions that are not fully aligned with the sport-specific demands of rhythmic gymnastics. Moreover, most balance assessments are conducted under conditions without additional motor or cognitive tasks and therefore fail to reflect the actual demands of rhythmic gymnastics, in which athletes must simultaneously maintain balance, manipulate apparatus, and respond to musical and spatial cues. Consequently, balance tests designed to assess performance under conditions characteristic of rhythmic gymnastics, particularly those incorporating combined motor and cognitive tasks, remain scarce and, to the best of our knowledge, effectively nonexistent. Although sport-specific movements of rhythmic gymnasts, which involve frequent perturbations of postural stability, represent a key component of performance, they are not the sole challenge present during competitive routines and their evaluations. In addition to high motor demands, rhythmic gymnasts are simultaneously exposed to substantial cognitive load resulting from the complex manipulation of various apparatuses (e.g., rope, hoop, ball, clubs, and ribbon) performed in synchrony with musical accompaniment. This combination of demands markedly increases cognitive and motor load, thereby complicating the maintenance of stable balance owing to the increased volume of information that the neuromotor system must simultaneously perceive and process.

Previous research has shown that balance ability is an important determinant of performance in rhythmic gymnastics. Multivariate analyses indicate that both static and dynamic balance significantly contribute to competition scores, with specific balance tests showing meaningful individual effects. Overall, balance has been identified as a significant predictor of performance, explaining a substantial proportion of performance variance in rhythmic gymnasts (approximately 24–35%) [9]. Flexibility, strength, agility, muscular endurance, balance, and specific coordination represent fundamental motor abilities that largely determine performance in rhythmic gymnastics from an early age. Higher-level gymnasts consistently achieve better results across these domains, confirming their key role in successful performance [10,11,12,13,14,15]. At the same time, each specific balance required for the successful execution of elements in rhythmic gymnastics demands an adequate level of these abilities, including well-developed general balance, muscular stability and endurance, as well as a high level of flexibility. Due to their interrelated nature and importance for performance, it is essential to develop and implement specific balance tests that integrate these capacities, enabling more precise assessment and more effective guidance of the training process [16].

Given that the fundamental objective of any purposeful kinesiological training and transformation process is the valid and reliable assessment of motor and functional abilities directly involved in sports performance, there is a clear need to develop new sports-specific testing protocols. Such protocols should enable a precise evaluation of the functional status of athletes, specifically rhythmic gymnasts, under conditions that replicate the real demands of their discipline. Accordingly, the primary aim of the present study was to construct and validate a novel sport-specific balance test that incorporates combined motor and cognitive demands.

## 2. Materials and Methods

### 2.1. Participants

The study included 12 girls, rhythmic gymnasts from the Rondo Rhythmic Gymnastics Club in Osijek, with a mean age of 9 ± 0.70 years. All participants and their legal guardians were informed in advance about the research protocol, and prior to the start of the study, an information sheet and informed consent form were provided to the parents or legal guardians of the gymnasts. All participants with signed consent forms were included in the study.

### 2.2. Study Design and Testing Procedure

The investigation was conducted in two distinct phases. The initial phase involved familiarization with the newly developed assessment, followed by baseline measurements of anthropometric variables, specifically body height and weight, as well as motor variables.

The first motor test administered to the participants was the Y Balance Test. The test was included as a validated measure of dynamic postural control and balance performance, demonstrating good to excellent intra-rater reliability (ICC = 0.85–0.91), as well as confirmed construct and discriminant validity in various athlete populations for the assessment of muscle control and functional stability [17]. Functional assessment of balance and stability that evaluates body control in three directions: anterior (forward), posteromedial (backward and toward the midline), and posterolateral (backward and outward). Participants stood barefoot on one leg at the center of a specialized Y-shaped platform (Functional Movement Systems, Inc., Chatham, VA, USA), while the other leg was extended and moved in one of the three directions, measuring the distance the leg could reach without losing balance or touching the ground with the other leg. Each direction was repeated three times, and the best results were recorded [18]. Given the specificity of training tasks, in which rhythmic gymnasts frequently maintain balance on a single leg, this test closely reflects the demands of real sport-specific situations.

Subsequently, measurements were taken using the modified Flamingo test with both eyes closed and eyes open. The test demonstrates good to excellent reliability (ICC = 0.84–0.98), acceptable interval of standard measurement error (SEM = 0.5–1.5), and confirmed construct and discriminant validity for assessing balance in children. Participants stood upright on dominant leg on the forefoot, with hands on the hips, while the other leg was lifted and bent at the knee so that the foot touched the knee of the supporting leg. The test was conducted under two conditions: with eyes open and eyes closed, during which the duration the participant could maintain stability without moving the lifted leg or losing balance was recorded. Each participant completed five trials, and the final score was calculated as the arithmetic mean of the three middle values, excluding the best and worst results [19].

Given the importance of range of motion, particularly at the hip joint, for maintaining postural alignment and dynamic balance, it is reasonable to assess the extent to which it also influences the newly developed test [20]. The flexibility assessment was conducted using the Sit and Reach test. Participants sat on the floor with legs fully extended, feet firmly against a box, and reached forward with hands together, palms facing down. They slowly bent at the waist, attempting to reach as far as possible toward the toes while keeping the knees straight. The farthest point reached by the hands was measured in centimeters and used to evaluate flexibility. The test was performed three times, with the best attempt recorded as the final score [21]. Assessment of trunk isometric strength was conducted using the plank test.

The plank test was included as a validated field-based measure of isometric trunk muscle endurance, which is one of the most important components of core stability. Previous research has demonstrated high reliability, with an intraclass correlation coefficient (ICC = 0.99). In addition, electromyographic analysis has confirmed substantial activation of trunk musculature during the test, further supporting its validity. Although the plank test does not directly assess balance, trunk muscle endurance contributes to postural control and body stability. Therefore, the test is theoretically justified as an indirect indicator of neuromuscular control related to balance performance. Participants assumed a position in which the forearms and toes were in contact with the ground, elbows positioned directly under the shoulders, and the body forming a straight line from head to heels. The objective was to maintain a straight alignment of the shoulders, hips, and legs for as long as possible without allowing the hips to drop or the body to sag. The duration was measured in seconds [22].

And finally, lateral hopping test was used as an indirect measure because it requires continuous control of the center of mass during rapid and repetitive lateral movements. Although classified as an agility task, successful performance depends on neuromuscular control, postural stability, and the ability to maintain balance during single-leg landings and quick transitions between support phases. Therefore, the test reflects dynamic balance control under sport-specific movement demands, particularly in activities requiring frequent changes in direction and unilateral stability. Side hop test is performed with the participant standing on one leg and hands placed on the hips. The task is to jump laterally back and forth between two parallel lines spaced 30 cm apart for 30 s, aiming to complete as many correct hops as possible. A hop is considered incorrect if the foot lands on the line or if the non-supporting leg touches the ground. Throughout the test, the participant must keep their hands on the hips. The total number of correctly executed hops is recorded for each participant [23].

**Figure 1:**
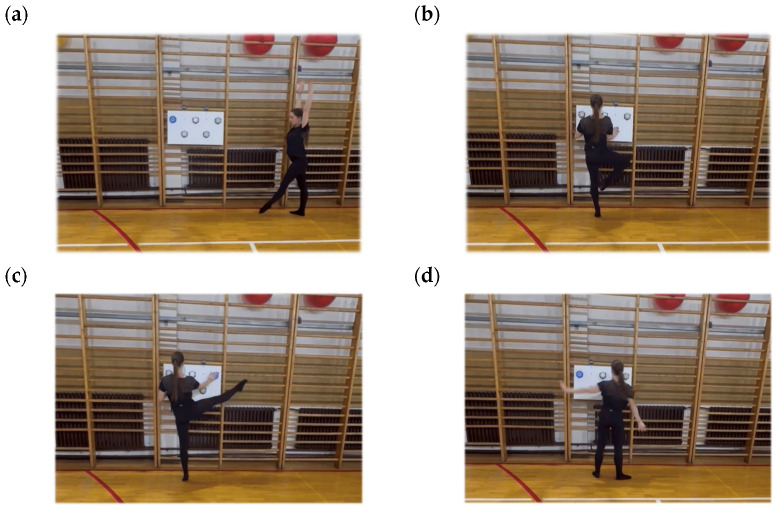
Scheme of testing procedure. (**a**) The participant stands sideways to the wall and prepares to perform a forward cartwheel; (**b**) after landing on the dominant leg, she assumes a stable full-foot stance, flexes the knee, and simultaneously rises onto the forefoot; (**c**) she performs a cognitive-motor task by pressing the lights in front of her while simultaneously fully extending the other leg to the side; (**d**) the test is completed once the heel of the supporting leg touches the ground.

On the subsequent day, specific balance was evaluated using the newly constructed test designed for rhythmic gymnasts. The second phase commenced on the tenth day following the initial specific balance evaluation, during which a retest measurement was conducted adhering to the same protocol, employing the newly devised balance test for rhythmic gymnasts. The tests were conducted in a fixed order for all participants to ensure standardized conditions and to minimize the influence of fatigue on subsequent tests. The assessment was carried out using a single tablet equipped with the MyJump 3 (My Jump Lab, Madrid, Spain) installed on Ipad 7 (Apple. Inc., Cupertino, CA, USA)application and five BlazePod™ light devices (Play Coyotta Ltd., Tel Aviv, Israel). The participants assumed an initial position facing sideways to the wall. Upon the examiner’s signal, she executed a cartwheel toward her dominant side and immediately upon landing, rose onto the tiptoes of the dominant leg and commenced the cognitive motor task. The task was predicated on responses to visual stimuli: when one of the BlazePod lights was randomly activated, the participant was required to touch the illuminated device as quickly as possible with her hand, maintaining contact for the shortest possible duration, while sustaining a single-leg stance on tiptoes and concurrently executing hip abduction with a fully extended knee of the contralateral leg. The gymnast is required to maintain a single-leg balance position for as long as possible while simultaneously performing the previously described tasks. The duration of the test was meticulously recorded in seconds, commencing from the moment the heel was elevated off the ground and the tiptoe position was attained until the heel of the supporting leg re-established contact with the ground, at which point the test was deemed complete. The time for each attempt was accurately documented using the MyJump application. The test was performed five times. The final outcome was computed as the mean value of the three measurements, excluding the most and least advantageous results. All participants undertook five consecutive trials in succession until the completion of the first round by all participants. The entire procedure was subsequently repeated two additional times, thereby ensuring that each participant completed three rounds of measurements. The final score was determined as the average of the results from all three rounds of voting. A schematic representation of the test procedure is presented in Figure 1 below (for video presentation please see the Supplementary Material).

### 2.3. Variables

Body height (BH) was recorded using a SECA 213 anthropometer (Seca GmbH, Hamburg, Germany), which offers precision to 0.1 cm. Measurements of body weight (BW) and body composition, such as body fat percentage (BF), muscle mass (MM), and body mass index (BMI), were obtained with an Omron BF-511 device (Kyoto, Japan), accurate to 0.1 kg. The Balance Test for Rhythmic Gymnasts (BTRG), a newly developed tool, was used to assess specific balance. To determine construct validity, BTRG results were compared with established balance tests, including the modified Flamingo Test on the dominant leg with eyes open (MFTOE) and closed (MFTCE), as well as the Y Balance Test (YBT), along with the additional assessments like Front Plank Test (FPT) and Sit and Reach test (SRT), to explore the theoretical framework of balance as measured by the BTRG.

### 2.4. Statistical Analysis

Descriptive statistics were used to summarize the results of the newly developed test, including the mean (M) and standard deviation (SD). Data normality was assessed using the Shapiro–Wilk test. Reliability was evaluated using both relative and absolute measures: relative reliability was determined using the test–retest procedure and the intraclass correlation coefficient (ICC_3,1_), while absolute reliability was assessed using the coefficient of variation (CV) and the standard error of measurement (SEM). The differences between the participant groups in the test results were analyzed using independent *t*-test. The construct validity was examined using factor analysis to identify the latent dimensions of motor abilities that primarily explain the test results. Pearson’s correlation coefficient was used to assess the relationships between the motor tests and the newly constructed specific balance test. Statistical significance was set at *p* < 0.05.

## 3. Results

Results in Table 1 indicate that the sample was relatively homogeneous in terms of age and anthropometric characteristics. The scores obtained in the specific balance test (BTRG) and its repeated measurement (BTRGR) were very similar, confirming the stability and repeatability of test performance.

As shown in Table 2, the test–retest analysis revealed an exceptionally high intraclass correlation, indicating excellent relative reliability of the test. Low values of the coefficient of variation and standard error of measurement further support the high absolute reliability of the newly developed instrument.

Table 3 reveals a statistically significant difference in the results of specific balance tests among gymnasts from various competitions. The main differences between gymnast in Program A and Program B are reflected in training load and the difficulty of performed elements. Gymnast in Program A have a higher weekly training volume (20 h per week) compared to those in Program B (12 h per week). In addition, gymnasts in Program A perform routines and elements with higher initial difficulty values during both training and competition, characterized by greater technical and performance complexity. Athletes participating in the advanced program demonstrated superior performance, thereby affirming the test’s discriminative capability.

In Table 4, the principal components method was used for factor extraction. The table also demonstrates that the factor analysis identified the first component as accounting for more than half of the total variance, while the first two components collectively explained over three-quarters of the variance. This structure might suggests the existence of a predominant latent dimension associated with balance and postural control in the rhythmic gymnast. To further explore the underlying structure and the relationships between the measured variables, the analysis proceeded to examine factor loadings after rotation (Table 5). Table 5 shows that the highest factor loadings on the first component were observed for the BTRG and the Y Balance Test, supporting the exploratory findings that the newly developed test may preliminarily assess the same construct of specific dynamic balance. A quartimax rotation was used in the analysis to aid interpretability of the factor structure. The remaining tests exhibited moderate to low loadings, further substantiating the construct validity of the new measurement instrument.

**Figure 2:**
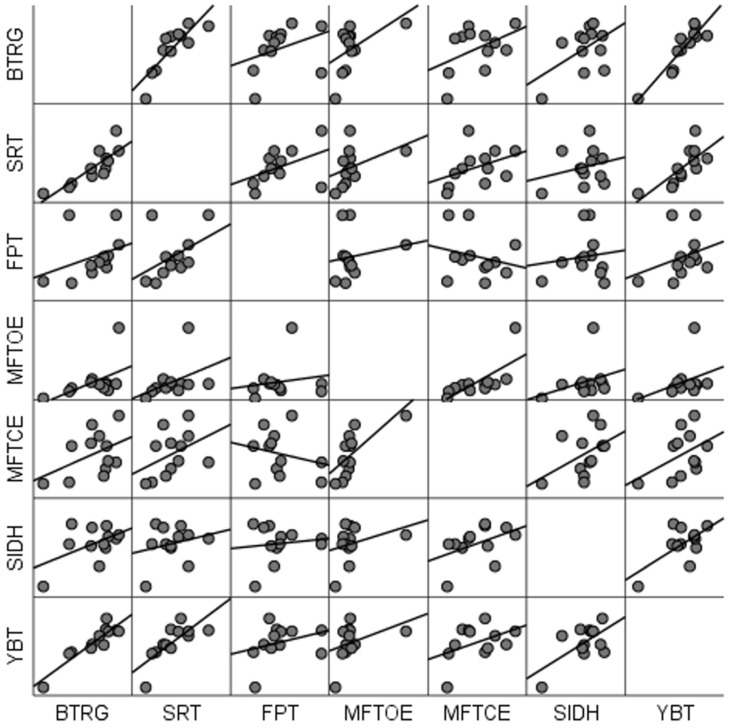
Legend: BTRG—newly specific balance for rhythmic gymnast; YBT—Y balance test; MFTCE—modified Flamingo test performed with closed eyes; SRT—sit and reach test; MFTOE—modified Flamingo test performed with the opened eyes; SIDH—side hop test; FPT—forward plank test.

Given the exploratory nature of the study, alternative and more descriptive statistical approaches were considered. Specifically, pairwise relationships between variables were explored using a scatter plot matrix, which allows for a clear visual inspection of the associations between all motor test variables and provides insight into potential linear and non-linear patterns without imposing strong distributional assumptions. Scatter plot is presented in Figure 2.

Table 6 presents the Pearson correlation coefficients, offering a preliminary and exploratory overview of the relationships between variables and the potential influence of specific motor abilities on the newly constructed test in rhythmic gymnasts.

## 4. Discussion

The results of this pilot study point to several important conclusions. One of the key preliminary findings was that the newly constructed sport-specific balance test for rhythmic gymnasts potentially demonstrated a very high level of reliability. Relative reliability assessed by the intraclass correlation coefficient (ICC = 0.96), together with indicators of absolute reliability (CV = 6.4% and SEM = 0.18), indicated high stability of results between the two repeated measurements of the observed tests. By comparing the obtained results with previous studies that have addressed the construction and validation of sport-specific testing protocols, it can be suggested that the testing protocol used in this study exhibited very satisfactory metric characteristics of reliability [24,25,26]. This level of reliability is particularly important given that the study was conducted on a sample of children with a mean age of 9.86 years old. Considering the specific characteristics of growth and development at this age, it is evident that balance development in children of this age is essential [27]. As rhythmic gymnastics requires early specialization compared to many other sports activities [28], it is necessary to have comprehensive and appropriate testing instruments that allow the assessment of complex motor and cognitive-motor demands, including apparatus manipulation and movement execution accompanied by music. A test that integrates almost all of these tasks within a single protocol represents a significant contribution to the assessment of sport-specific abilities in rhythmic gymnastics. One of the most important characteristics of a test’s discriminative validity is its ability to differentiate participants according to their quality or performance level [24]. The results of the independent *t*-test presented in Table 3 may indicate that rhythmic gymnasts enrolled in the more advanced A competitive program achieved significantly better results, that is, a longer ability to maintain sport-specific balance (BTRG = 3.38 ± 0.28), compared to gymnasts from the B competitive program (BTRG = 1.96 ± 0.81). The observed difference of 1.42 s in favor of the A program participants was statistically significant at *p* < 0.01. In addition to potentially confirming the satisfactory metric characteristics of validity, this finding has important practical implications. Primarily, the test may reflect meaningful differences in balance performance between athletes of different competitive levels, with evidence indicating superior balance control in more elite rhythmic gymnasts [16]. This suggests that balance performance could be considered a potential indicator of athletic potential; however, it should not be interpreted as a standalone measure of talent identification, as talent development is a multidimensional construct influenced by a range of additional factors such as training experience, maturation, and psychological characteristics. At a stage when more intensive selection and athlete orientation begin, a testing protocol that objectively assesses the ability to maintain sport-specific balance under a cognitive-motor load may represent a valuable tool within the selection system. Gymnasts who demonstrate superior sensorimotor integration and more effective body control under load allow for more precise and objective planning of their athletic development. At the same time, early sports specialization should be approached with caution, as overly intensive and inadequately structured training processes may negatively affect the psychological state of young athletes and potentially lead to increased stress, early sports dropout, or an elevated risk of injury, which can further complicate the overall athlete development process [29]. Table 4 and Table 5 present the results of the factor analysis conducted to determine the construct validity of the newly developed test. Factor analysis was used to identify which latent motor dimensions, and to what extent, explained performance on the new test. Table 4 presents the initial eigenvalues and their corresponding communalities. According to the Kaiser criterion, which is one of the most commonly used rules for factor retention in factor analysis and principal component analysis, only factors with eigenvalues greater than 1 are retained, as they explain a greater amount of variance than a single original variable [30]. In accordance with this criterion, two factors were extracted (first factor = 3.80; second factor = 1.39), together accounting for 74.22% of the total variance of the tests. The factor loading analysis in Table 5 shows that the BTRG (0.89) and YBT (0.86) tests have high loadings on the first component, indicating a dominant latent dimension. Given previous findings that clearly demonstrate that the Y Balance Test primarily measures the latent motor dimension of balance [31], it can be suggested that the newly constructed BTRG also demonstrates satisfactory construct validity. In conclusion, the preliminary findings of the first extracted component could possibly be interpreted as the rhythmic gymnast’s ability to effectively integrate information from the somatosensory, vestibular, and visual systems to maintain stability, that is, sport-specific balance, during the execution of complex motor tasks. Furthermore, the correlation analysis preliminarily and exploratorily indicated that the variables YBT and SRT showed significant associations with the newly constructed test. These tests primarily reflect balance and flexibility capacities. These findings are in line with previous research highlighting the potential role and importance of flexibility in balance performance [10], suggesting that adequate flexibility may facilitate the execution of balance related tasks and contribute to more efficient postural control. In this context, flexibility may represent one of the underlying capacities associated with performance in the newly constructed test, although such interpretations remain preliminary and should be treated with caution. Although exploratory in nature, these findings may suggest that such motor abilities could have a latent influence on performance in the newly constructed test.

### Study Limitations

The primary limitation of this study was its small sample size. Although this research was clearly defined as a preliminary pilot study, a larger sample is required to perform a more robust factor analysis and to enable generalization of the results to a broader population of rhythmic gymnasts. Furthermore, it would be desirable to extend the study to a more heterogeneous age sample, which would allow the assessment of balance in older age categories, such as junior and senior gymnasts. Additionally, potential dependence on technological resources should not be overlooked, as the test protocol is implemented using an application and a specific visual system, which may represent a limitation for clubs with fewer resources.

## 5. Conclusions

The recently developed Balance Test for Rhythmic Gymnasts (BTRG) has potential to be a successful, reliable, and validated measurement tool. The test exhibits high reliability and stability of results, rendering it a consistent instrument for assessing the functional status of rhythmic gymnasts. Construct analysis has confirmed that the test might effectively measure the latent dimensions of specific balance in rhythmic gymnastics. Furthermore, the BTRG has potential for distinguishing between gymnasts of varying skill levels, with those in advanced programs achieving superior results compared to those in standard programs. The value of this test lies in its sport-specific approach. Unlike general balance tests, the BTRG integrates cognitive-motor demands, including responses to visual stimuli while maintaining balance after an acrobatic element, thereby more accurately reflecting real competition conditions. Due to its potential in validity and sensitivity, the test might be recommended to coaches as a valuable tool for evaluating and monitoring athletes’ progress, as well as supporting talent identification and development. However, the authors noted that the limited number of participants in the pilot study was a limitation and suggested that future research should include a larger sample to further confirm the test’s applicability in a broader context.

## Figures and Tables

**Table 1:** Basic anthropometric measurements, normality of distribution and performance outcomes on a specific balance and motor test.

Variable	Mean ± SD	Shapiro–Wilk Test	*p*
AGE (years)	9.86 ± 0.70		
BH (cm)	144.98 ± 9.32		
BW (kg)	35.62 ± 8.07		
BMI (kg/m^2^)	16.89 ± 2.68		
BTRG (s)	2.80 ± 0.89	0.94	0.44
BTRGR (s)	2.79 ± 0.90	0.88	0.11
SRT (cm)	57.62 ± 7.06		
FPT (s)	128.04 ± 43.92		
MFTOE (s)	195.52 ± 141.96		
MFTCE (s)	47.80 ± 34.48		
SIDH	55.33 ± 9.67		
YBT (cm)	215.75 ± 38.85		

Legend: BH—body height; BW—body weight; BMI—body mass index; BTRG—first measure of newly specific balance test for rhythmic gymnast; BTRGR—newly specific balance retest for rhythmic gymnast; SRT—sit and reach test; FPT—forward plank test; MFTOE—modified Flamingo test with open eyes; MFTCE—modified Flamingo test with closed eyes; SIDH—side hoop test measured with correct number of repetition; YBT—Y balance test; *p*—statistical significance.

**Table 2:** Results of the first and second measurements of the balance among with reliability metrics.

	1st Test	2nd Test (Retest)	ICC_3,1_ (95% CI)	CV%	SEM
BTRG (s)	2.80	2.79	0.96 (0.87–0.99)	6.4	0.18

Legend: BTRG—results of first and second measurement of newly specific developed test; ICC_3,1_—intraclass coefficient of correlation; CI—interval of confidence; CV—coefficient of variation; SEM—standard error of measurement.

**Table 3:** Test results according to the type of competition program.

	Program	Mean ± SD	Mean Difference	*p*	Cohen’s d
BTRG (s)	A (*n* = 7)	3.38 ± 0.28	1.42	0.01	2.55
	B (*n* = 5)	1.96 ± 0.81			

Legend: BTRG—newly specific balance test for rhythmic gymnast; SD—standard deviation; *p* level of significance; A—competitors of the advanced rhythmic gymnastics program; B—competitors of the regular rhythmic gymnastics program; *p*—level of statistical significance; Cohen’ d—effect size magnitude; values of 0.2, 0.5, and 0.8 indicate small, medium, and large effects, with values > 1.2 interpreted as very large effects.

**Table 4:** Initial eigenvalues and explained variance.

	Initial Eigenvalues	
Total	% of Variance	Cumulative %
3.80	54.32	54.32
1.39	19.90	74.22
0.83	11.83	86.05
0.63	9.00	95.06
0.22	3.10	98.16
0.87	1.24	99.14
0.42	0.60	100.00

**Table 5:** Component matrix.

	Components	
	1	2
BTRG	0.89	0.33
YBT	0.86	0.30
MFTCE	0.77	−0.55
SRT	0.76	0.45
MFTOE	0.73	−0.24
SIDH	0.66	−0.07
FPT	0.21	0.84

Legend: BTRG—newly specific balance for rhythmic gymnast; YBT—Y balance test; MFTCE—modified Flamingo test performed with closed eyes; SRT—sit and reach test; MFTOE—modified Flamingo test performed with the opened eyes; SIDH—side hop test; FPT—forward plank test.

**Table 6:** Pearson correlation matrix of motor variables and the newly constructed test.

r	BTRG
BTRG	1.00
YBT	0.91 **
SRT	0.84 **
FPT	0.35
MFTCE	0.45
MFTOE	0.52
SIDH	0.50

Legend: r—Pearson’s coefficient of correlation; **—correlation is significant at the 0.01 level; BTRG—newly specific balance for rhythmic gymnast; YBT—Y balance test; MFTCE—modified Flamingo test performed with closed eyes; SRT—sit and reach test; MFTOE—modified Flamingo test performed with the opened eyes; SIDH—side hop test; FPT—forward plank test.

## Data Availability

The data presented in this study are available on request from the corresponding author.

## Supplementary Materials

The following supporting information can be downloaded at: https://www.mdpi.com/article/10.3390/sports14050178/s1, Video S1: Newly developed test.
